# Target-Following Control of a Biomimetic Autonomous System Based on Predictive Reinforcement Learning

**DOI:** 10.3390/biomimetics9010033

**Published:** 2024-01-04

**Authors:** Yu Wang, Jian Wang, Song Kang, Junzhi Yu

**Affiliations:** 1 Department of Automation, Tsinghua University, Beijing 100084, China; yu_wang@tsinghua.edu.cn; 2 The Laboratory of Cognitive and Decision Intelligence for Complex System, Institute of Automation, Chinese Academy of Sciences, Beijing 100190, China; jianwang@ia.ac.cn (J.W.); song.kang@ia.ac.cn (S.K.); 3 The School of Artificial Intelligence, University of Chinese Academy of Sciences, Beijing 100049, China; 4 The State Key Laboratory for Turbulence and Complex Systems, Department of Advanced Manufacturing and Robotics, College of Engineering, Peking University, Beijing 100871, China

**Keywords:** biomimetic motion, biomimetic autonomous system, target following, deep reinforcement learning, predictive control

## Abstract

Biological fish often swim in a schooling manner, the mechanism of which comes from the fact that these schooling movements can improve the fishes’ hydrodynamic efficiency. Inspired by this phenomenon, a target-following control framework for a biomimetic autonomous system is proposed in this paper. Firstly, a following motion model is established based on the mechanism of fish schooling swimming, in which the follower robotic fish keeps a certain distance and orientation from the leader robotic fish. Second, by incorporating a predictive concept into reinforcement learning, a predictive deep deterministic policy gradient-following controller is provided with the normalized state space, action space, reward, and prediction design. It can avoid overshoot to a certain extent. A nonlinear model predictive controller is designed and can be selected for the follower robotic fish, together with the predictive reinforcement learning. Finally, extensive simulations are conducted, including the fix point and dynamic target following for single robotic fish, as well as cooperative following with the leader robotic fish. The obtained results indicate the effectiveness of the proposed methods, providing a valuable sight for the cooperative control of underwater robots to explore the ocean.

## 1. Introduction

With the rapid development of science and technology, the field of biomimetics has witnessed substantial advance in recent years, garnering widespread attention from researchers globally. With a long history of natural evolution, biological fish have acquired remarkable motion capabilities. They demonstrate proficiency in executing various acrobatic maneuvers, including rapid start and stop, inter-media leaping, and other sophisticated actions. Additionally, natural fish can achieve hydrodynamic drag reduction and energy efficiency through the utilization of diverse biological mechanisms.

Inspired by nature, the biomimetic robotic fish has attracted the attention of many scientists and engineers [1,2,3,4,5,6]. Katzschmann et al. designed a new kind of soft robotic fish named Sofi, which can interact with remote control personnel through ultrasonic communication module and realize the close observation of underwater organisms with a maximum diving depth of 18 m [1]. By mimicking yellowfin tuna, White et al. developed a series of high-speed biomimetic robotic tuna [7,8], and achieved tail-fin flapping frequencies of up to 15 Hz. To quantify the role of body flexibility in high-speed swimming, they further presented Tunabot Flex, which could achieve a high swimming speed of 4.6 BL/s (body length per second, BL/s) at a swing frequency of 8 Hz. Yu et al. designed a robotic dolphin. Based on the motion control strategies, a high swimming speed of 2.05 m/s (2.85 BL/s) was achieved, and the action of repetitive leaping similar to that of biological dolphins was completed [9]. Compared with the traditional autonomous underwater vehicles (AUVs), the biomimetic robotic fish has the advantages of high maneuverability, strong concealment, and better biocompatibility. These attributes underscore their considerable potential for application across diverse domains.

More importantly, by learning the motion mechanism of biological fish, the movement performance of the robotic fish can be further improved, e.g., by using fish schooling movement to save energy. It is hypothesized that fish schooling movements can improve the hydrodynamic efficiency via a certain swimming mode [10]. In a school of fish, the leader fish tend to consume more energy than the follower fish. The eddy current generated by the tail of leader fish can provide a certain amount of water power to the follower, thus achieving the effect of energy saving. This movement has a similar phenomenon in birds. As a result, biological fish tend to travel in flocks for long-distance voyages. Many researchers have investigated the fish schooling mechanism. Li et al. focused on how the fish plan their movement to save energy and achieve larger thrust from the vortices generated by others [11,12]. They designed some bionic robotic fish to measure the energy consumption when the robotic fish swim in the pool. Further, a vortex phase-matching strategy was obtained, indicating that the schooling fish exhibited a tailbeat phase difference that varied linearly with front–back distance. They also found that when the fish swim side by side, an individual could improve its efficiency if they changed the tailbeat phase to a certain angle, such as 0.25 π . By measuring the actual movement of 15 fish, Marras et al. found that compared with the individual swimming, the schooling fish in any position can save energy, and the fish swimming behind the neighbours showed the best performance [13]. Thandiackal et al. conducted an interesting experiment to observe the movement of natural trout when it interacted with the thrust wakes via a robotic mechanism. The results illustrated that the trout exhibited reduced pressure drag, further proving the energy saving [14]. Li et al. investigated the pressure and vorticity fields between a single fish and a pair of fish, and offered some results. However, there are insufficient conclusions about motion benefits of fish schooling [15]. Dai et al. investigated a variety of stable formations with the schooling of two, three and four self-propelled fish-like swimmers, and examined the energy efficiency of each formation [16]. Verma et al. explained the energy-saving mechanism in the schooling behavior of fish. By combining deep reinforcement learning and fluid simulation, an energy-saving strategy was proposed, which enabled followers to save energy by using vortices in the leader’s wake [17]. More studies can be found in [18].

With regard to the target following control, there are many related results. Dai et al. [19] designed a robust tube model predictive controller (MPC) with an extended Kalman filter target observer for an underwater vehicle-manipulator system, specifically tailored to address the challenge of capturing moving targets. Cui et al. [20] proposed an optimal trajectory tracking method for AUVs, applying reinforcement learning techniques with critic and action neural networks. He et al. [21] formulated asynchronous multithreading proximal policy optimization-based algorithms to tackle issues related to path planning and trajectory tracking in unmanned underwater vehicles. Jiang et al. [22] introduced a model-free attention-based, model-agnostic meta-learning algorithm for AUVs, demonstrating efficacy in achieving high-precision tracking tasks. Zou et al. [23] designed an image-guided motion controller which consists of a genetic algorithm-based linear quadratic regulator velocity controller and direction controller to realize mobile target following for micro-robotic swarms. Yan et al. [24] designed a reinforcement learning and orthogonal fractional factorial design-based tracking controller for AUVs to enhance the scalability of uncertainty evaluation. Shi et al. [25] applied a hybrid actors–critics architecture to improve following control accuracy of AUVs. Gao et al. [26] introduced a fixed-time resilient cooperative edge-triggered estimation and control framework designed to facilitate cooperative target tracking for unmanned surface vehicles (USVs). Wai et al. [27] constructed an adaptive following control scheme with a dynamic recurrent fuzzy neural network that allowed the vision-based mobile robot to track the moving target. Huang et al. [28] designed a homography-based visual servo controller that allowed the unmanned aerial vehicle to track the moving ship trajectory. Lin et al. [29] designed an image-based visual servoing geometric controller for quadrotors for tracking the desired trajectory.

Our work is motivated by the collective motion observed in fish schooling, where a phenomenon is known to enhance motion efficiency and reduce energy consumption. The studies on fish schooling movement can be theoretically categorized into kinematic and behavioral levels. At the kinematic level, the studies primarily focus on the macroscopic distances and swimming postures among fish. By closely observing the swimming patterns within fish schools, optimal distances and directions can be discerned. Further, at the behavioral level, greater emphasis may be placed on the individual body swimming postures of fish. For instance, in the context of tail-wagging fish swimming, it becomes imperative to synthesize parameters such as tail-wagging frequency, amplitude, and phase differences within the fish school. In this paper, we focus on the kinematic level, wherein we translate the complexities of fish schooling swimming into the pursuit of target position and attitude, thereby providing foundational support for biomimetic research at the behavioral level. The primary contributions of this paper can be concluded in three aspects.

- Inspired by fish schooling movement, we focus on the kinematic level and propose a target following control framework, including a predictive deep deterministic policy gradient controller (PDDPG) and nonlinear model predictive controller (NMPC).
- Aiming to address the hysteresis characteristics of following control for the robotic fish, we introduce the predictive concept into the deep deterministic policy gradient method. By predicting the future state and adding it to the buffer pool, we effectively mitigate the overshooting phenomenon during the tracking process. Furthermore, the state space is intentionally designed in a normalized manner, concurrently featuring a multi-objective optimization reward function.
- Taking the kinematic and dynamic models as the predictive model, we derive the nonlinear model predictive control law with full consideration of the stage cost and terminal cost. Extensive simulations are carried out to verify the effectiveness of the proposed PDDPG and NMPC methods.

The subsequent sections of this paper are structured as follows. Section 2 provides an exposition of the problem statement and the control framework. Section 3 delves into the target-following control methodologies, encompassing the predictive deep deterministic policy gradient controller and the nonlinear model predictive controller. Furthermore, Section 4 presents simulation results, followed by a comprehensive analysis. Finally, Section 5 offers concluding remarks to summarize the paper.

## 2. Problem Statement and Control Framework

In this section, we commence by introducing the target-following task and succinctly present the kinematic and dynamic models pertaining to the underwater robot. Subsequently, aligned with the specified task, we formulate a target-following framework for the robotic fish. Additionally, an alternative movement strategy is proposed, which integrates both the predictive deep deterministic policy gradient controller and the nonlinear model predictive controller.

**Figure 1:**
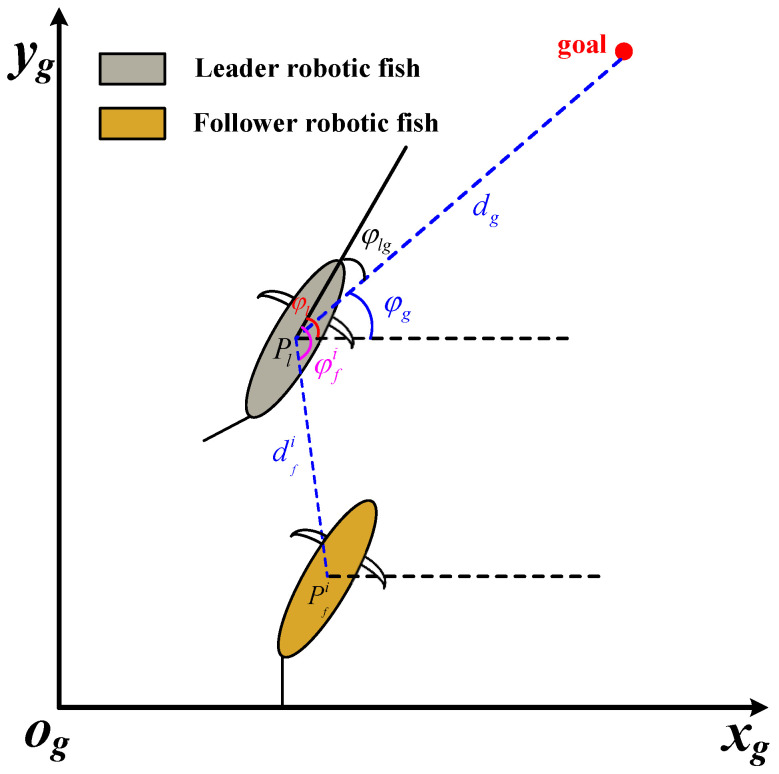
The illustration of the target-following task and coordinate system definition.

In view of the observations that fish schooling movement in nature can save energy consumption and improve movement efficiency, this paper aims to design a target-following control method for the robotic fish. As shown in Figure 1, by selecting a robotic fish as the leader, a path cruise task that involves setting target points can be executed. $P_{l}=\left(x_{l},y_{l}\right)$ denotes the real-time position of the leader robotic fish while $\varphi_{l}$ is its yaw angle. $d_{g}$ and $\varphi_{g}$ indicate the Euclidean distance and relative direction between the leader robotic fish and the target points, respectively. $\varphi_{lg}=\varphi_{l}-\varphi_{g}$ illustrates the attitude direction difference between the leader robotic fish and the target points.

Furthermore, we set some robotic fish as followers. $P_{f}^{i}=\left(x_{f}^{i},y_{f}^{i}\right)$ denotes the real-time position of the follower robotic fish, where i=1,2,…,n . The purpose of the following task is to maintain the set target distance $d_{f}^{i}$ and direction $\varphi_{f}^{i}$ between the follower and leader robotic fish. Given the aforementioned variables, the target position point for the follower can be obtained as follows:

(1) $$\begin{array}{c} x_{f}^{i}=x_{l}+d_{f}^{i}\cos\left(\varphi_{f}^{i}-\varphi_{l}\right)\\ y_{f}^{i}=y_{l}+d_{f}^{i}\sin\left(\varphi_{f}^{i}-\varphi_{l}\right) \end{array}.$$

It should be noted that the target point undergoes real-time variations with the movements of the leader. Consequently, for the follower, this task is characterized as a dynamic following mission. One of the principal improvements of this study lies in the approach based on a deep reinforcement learning framework. During training, static target-following scenarios are employed, yet the method is endowed with the capability to dynamically follow targets. Besides, for each single robotic fish, we offer the kinematic model for the following control, which can be formulated by

(2) $$\dot{x}=u\cos\varphi -v\sin\varphi ,$$

(3) $$\dot{y}=u\sin\varphi +v\cos\varphi ,$$

(4) $$\dot{\varphi }=r,$$

where $p\left(t\right)=\left(x\left(t\right),y\left(t\right),\varphi \left(t\right)\right)$ represent the position and yaw angle with respect to inertia frame, respectively. (u,v,r) denote the linear and angular velocities with respect to body frame, respectively. Thereafter, the dynamic model can be derived as follows:

(5) $$\dot{u}=\frac{1}{m_{11}}\left(\tau_{u}+m_{22}vr-d_{11}u\right),$$

(6) $$\dot{v}=\frac{1}{m_{22}}\left(-m_{11}ur-d_{22}v\right),$$

(7) $$\dot{r}=\frac{1}{m_{33}}\left(\tau_{r}+\left(m_{11}-m_{22}\right)uv-d_{33}r\right),$$

where $\left(m_{11},m_{22},m_{33}\right)$ and $\left(d_{11},d_{22},d_{33}\right)$ are the mass and damping parameters larger than zero. $\tau_{u}$ and $\tau_{r}$ indicate the thrust force and yaw moment, respectively.

**Figure 2:**
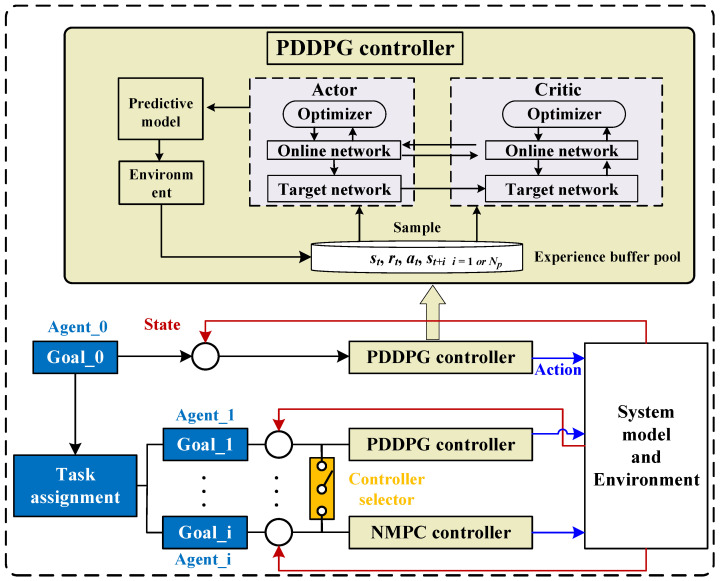
The target following control framework.

Therefore, based on the aforementioned problem statement, this paper proposes a target-following control framework, as illustrated in Figure 2. The framework comprises a biomimetic autonomous system consisting of one leader robotic fish (referred to as Agent_0) and multiple follower robotic fish. Initially, a cruising target point is set for the leader robotic fish. Utilizing the proposed PDDPG controller, real-time control force and moment can be generated to achieve cruising control. It is noteworthy that although these target points are discrete, if they are relatively close, an effect similar to continuous path following can be achieved. Subsequently, through a task allocation module based on the principles of natural fish, real-time target following positions can be determined for each follower robotic fish. Furthermore, for each follower robotic fish, a controller selector is designed, corresponding to the proposed PDDPG controller and NMPC, to output real-time control force and moment for target following. It is essential to emphasize the dual purposes of designing the selector. On one hand, the PDDPG controller exhibits strong environmental adaptability and scalability, suggesting that it has an advantage over NMPC when introducing random obstacle avoidance tasks in this mission. However, NMPC features stable solution finding and smooth motion output, contributing to improved control stability. On the other hand, in this paper, the two controllers are independent and are switched through a toggle switch. In practice, they can be organically integrated based on certain principles, such as event-triggered mechanisms, according to different task scenarios.

## 3. The Methodology of Target Following Control

### 3.1. The Predictive Deep Deterministic Policy Gradient Controller

In consideration of the dynamic characteristics and strong interference in underwater environments, this section introduces a following controller based on a predictive deep deterministic policy gradient. Firstly, the target-following problem is formulated as a multi-objective optimization issue through the design of network architecture, state space, action space, and reward function. Secondly, to enhance the training performance, normalization scaling is applied to the designed state variables, and reward values are standardized. More importantly, due to the highly nonlinear nature exhibited by biomimetic robotic fish, traditional control methods often result in following overshooting. To address this issue, the predictive approach is incorporated into the conventional deep deterministic policy gradient (DDPG) method. Specifically, when certain conditions are met, state variables and reward values after $N_{p}$ steps are calculated and stored in a buffer pool. During the testing stage, actions can be output from the network based on the state variables after $N_{p}$ steps. This technique can expand the training space to some extent, effectively avoiding overshooting without increasing the state space, thereby reducing network complexity.

First, the target-following task can be regarded as a Markov decision process. The tuple data comprises the state variable s∈S , the action variable a∈A , the reward function R(s) , the state transition function $F:\left(s,a\right)\to s^{\prime }$ , and the discount factor γ . By inputting the current state into a neural network, the action variable can be outputted, leading to the transition to the next state. The optimization objective is to maximize the reward function, driving parameter updates in the neural network. In recent years, DDPG has come to stand out as a reinforcement learning algorithm that has garnered substantial interest in recent years due to its efficacy in addressing challenges associated with continuous action space problems. Its applications span diverse domains, including robotics, control, and various other fields. DDPG amalgamates concepts from both value-based and policy-based reinforcement learning, utilizing a dual-neural network architecture comprising the Critic and the Actor. In this paper, we introduce certain enhancements to the conventional DDPG framework to accomplish the target-following task.

For deep reinforcement learning algorithms, the selection of appropriate state and action spaces, along with the design of a suitable reward function, stands as a pivotal determinant of network performance. In the subsequent section, specific design methodologies will be elucidated.

#### 3.1.1. State Space

In pursuit of reducing the complexity of deep neural networks, we exclusively focus on the design of two state variables as follows:

-
$d_{sg}$ : The state variable considered in this context pertains to the distance between the current position of the robot and the target point. This variable primarily ensures that the robot consistently approaches the target point at a predetermined velocity.
-
$\varphi_{sg}$ : The state variable involves the angular separation between the robot’s current position and the target point. This variable is crucial for ensuring the robot’s sustained alignment towards the target, serving the dual purpose of minimizing travel distance and maintaining a stable motion posture.

To enhance training performance and expedite convergence, we propose a normalization and scaling technique for a specific set of state variables. Firstly, we determine the maximum values for two state variables. In this study, the Euclidean distance between the robot’s starting point $\left(x_{o},y_{o}\right)$ and the target point $\left(x_{g},y_{g}\right)$ is designated as the maximum value for $d_{sg}$ , as follows:

(8) $$d_{\max}=\sqrt{{\left(x_{o}-x_{g}\right)}^{2}+{\left(y_{o}-y_{g}\right)}^{2}}.$$

As for $\varphi_{\max}$ , we set it to 2 π . Additionally, after normalization, we introduce a scale-up factor for two primary purposes. Firstly, post-normalization, the values fall within the [0, 1] range, which might be unsuitable for network convergence. Hence, amplification is applied. Secondly, considering the difference in the physical interpretations of $d_{sg}$ and $\varphi_{sg}$ , it is necessary to balance their magnitudes for faster convergence when inputting into the network. It is noteworthy that the selection of $d_{\max}$ is not a fixed value due to the real-time variability of the follower robot’s target. Hence, by dynamically updating the target point $\left(x_{g},y_{g}\right)$ for the follower robot, more effective action values can be obtained. Considering these aspects, the formulated state variables are expressed as follows:

(9) $$\begin{array}{c} {\tilde{d}}_{sg}=k_{1}\frac{d_{sg}}{d_{\max}}\\ {\tilde{\varphi }}_{sg}=k_{2}\frac{\varphi_{sg}}{\varphi_{\max}} \end{array}.$$

#### 3.1.2. Action Space

Based on the kinematic and dynamic models of biomimetic robotic fish, we define forward thrust and yaw moment as action variables. In contrast to conventional kinematic navigation approaches, we directly employ control quantities as actions, implying that it is necessary to traverse two layers of non-linear models, namely kinematics and dynamics, which increases the learning complexity. Additionally, building upon our previous work, we set the action ranges for forward thrust and yaw torque to [0, 6 N] and [−6 Nm, 6 Nm], respectively. Moreover, since this study does not involve information exchange among robot swarms, the leader robotic fish does not decelerate when the distance between the leader and follower robotic fish is substantial. Hence, it is essential to ensure that the follower robotic fish possesses the ability to catch up with the leader, leading to the specification of the maximum forward thrust range for the follower robotic fish as [0, 8 N].

Furthermore, the forward thrust of the robotic fish is constrained to be consistently greater than zero, while the yaw torque exhibits bilateral symmetry. Therefore, a bilateral correction is applied to the forward thrust. Specifically, during both the network output and replay buffer storage phases, the range of forward thrust is adjusted to [−3 N, 3 N]. When inputted into the training environment, the forward thrust outputted by the network is increased by 3 N, rendering it unilaterally positive, and subsequently fed into the motion model to update the environmental information.

#### 3.1.3. Reward Function

The reward function constitutes a pivotal element in deep reinforcement learning. With full consideration of path smoothness and length factors, a multi-objective optimization reward function is proposed as follows:

(10) $$R=\sum_{i=1}^{3}c_{i}r_{i},$$

where $c_{i}$ denotes the weight coefficients and $r_{i}$ represents the different reward forms. There are three principles.

- The principle of minimum distance: it is expressed as $r_{1}=-\left|{\tilde{d}}_{sg}\right|$ , primarily employed to minimize the length of the motion path.
- The principle of directional convergence: it is expressed as $r_{2}=-\left|{\tilde{\varphi }}_{sg}\right|$ , with the aim of guiding the robot to orient itself towards the target point during motion. This principle not only contributes to the reduction in path length but also serves to ensure a certain degree of stability in the output yaw moment.
- The path-smoothing principle: it is characterized by $r_{3}=-\left|{\tilde{\varphi }}_{sg}-{\tilde{\varphi }}_{sg}^{\prime }\right|$ , where ${\tilde{\varphi }}_{sg}^{\prime }$ represents the previous time step’s ${\tilde{\varphi }}_{sg}$ . This principle primarily aims to enhance control stability by minimizing the yaw angle difference between consecutive time steps, thereby smoothing the motion path.

#### 3.1.4. Predictive Concept-Based Improvement

In light of the aforementioned state space, action space, and reward function, it is evident that the learning objective of the proposed method is to achieve rapid and smooth target following. However, in practical tracking scenarios, due to the highly nonlinear characteristic of the system, the steering of robotic fish exhibits a certain degree of lag, leading to the occurrence of overshooting phenomena. To address this issue, the most direct solution is to introduce angular velocity as an additional state variable and incorporate it into the learning network. Nevertheless, this approach encounters two primary challenges. Firstly, the introduction of angular velocity increases the complexity of the state space, thereby escalating the training difficulty. Secondly, in real-world applications, angular velocity information is typically obtained from inertial measurement unit (IMU) sensor modules, making it susceptible to external environmental factors and noise, manifesting notable information instability such as abrupt fluctuations. Therefore, angular velocity is deemed unsuitable as a state variable. With these considerations, this paper integrates a predictive approach into the traditional DDPG framework, presenting a novel training architecture. This structure effectively mitigates overshooting phenomena without introducing additional state variables.

In the conventional DDPG algorithm, the replay buffer stores a series of experience tuples $\left(s_{t},a_{t},s_{t+1},r_{t}\right)$ , including the representation of the state at the next time step $s_{t+1}$ , i.e., $s_{t+1}=f\left(s_{t},a_{t}\right)$ . Here, *f* denotes a composite motion model of kinematics and dynamics. The key improvement in the proposed method lies in the replacement of the current state with the state quantity obtained after $N_{p}$ steps when the steering angular velocity exceeds a predefined threshold. Both the state after $N_{p}$ steps and the current state are then stored in the replay buffer, i.e., $\left(s_{t},a_{t},s_{t+N_{p}},r_{t}\right)$ . This process generates a sequence of states as

(11) $$\begin{array}{c} S=\left(s_{t+N_{p}},s_{t+N_{p}-1},\cdots ,s_{t+1},s_{t}\right)\\ s_{t+i}=f\left(s_{t+i-1},a_{t}\right) \end{array}.$$

Based on the above illustration, the calculation process of PDDPG is presented in Algorithm 1.
**Algorithm 1** Algorithm for PDDPG
  1:Initialize the parameters of Actor network and Critic network.  2:Initialize the experience replay buffer pool.  3:**for** episode = 1 to *N* **do**  4:      Reset the control system, and obtain the initial states $s_{0}$ .  5:      **for** step =1 to *M* **do**  6:             According to the trained strategy, select the output action with the added noise information.  7:             Perform actions in the model environment.  8:             **if** $\dot{\varphi }>$ $20^{\circ }$ /s **then**  9:                   Apply the predictive model, and calculate $s_{t+N_{p}}$ .10:                  Put $\left(s_{t},a_{t},s_{t+N_{p}},r_{t}\right)$ into buffer pool.11:            **else**12:                  Based on the motion model, calculate $s_{t+1}$ .13:                  Put $\left(s_{t},a_{t},s_{t+1},r_{t}\right)$ into buffer pool.14:            **end if**15:            Sample a subset of data from the experience replay buffer for network updating.16:            Update the Critic network according to the loss.17:            Update the Actor network based on deterministic policy gradient followed by the target Actor network.18:     **end for**19:**end for**


### 3.2. The Nonlinear Model Predictive Controller

In recent years, the utilization of MPC has become increasingly prevalent within the domain of robotics. MPC proves valuable not only in tackling the intricacies of systems with multiple inputs and outputs, but also in addressing and managing control constraints effectively. The control approach employed in this paper can be considered as a means of achieving setpoint stabilization to a certain extent. This is achieved by designing a controller that effectively stabilizes a predefined stationary setpoint.

Although the approach proposed in this paper also allows for dynamic target points to be followed, the switching of these dynamic targets is governed by specific triggering rules. On one hand, for the leader robotic fish, the target point switching criterion is based on the condition that the robotic fish is within a certain threshold distance from the target. As a result, this type of target point switching can be considered as setpoint stabilization on a time scale. On the other hand, for the follower robotic fish, the target points change in real time with the position of the leader robotic fish. However, due to the time-independence characteristic of target point locations during the controller design process, these changes can be simplified as setpoint stabilization. Furthermore, in order to achieve the following control, we need to stabilize the key variables, including the planar position and the yaw attitude. Hence, based on the kinematic and dynamic models of the robotic fish, the state variables can be selected as $P_{f}=\left(x,y,\varphi \right)$ . Correspondingly, we consider the forward thrust and yaw moment as control variables, i.e., $u_{f}=\left(\tau_{u},\tau_{r}\right)$ .

This design implies the incorporation of both the kinematic and dynamic models as components of the predictive model, which simplifies the controller design process. However, the combination of two nonlinear models may introduce a degree of computational complexity and elevate optimization challenges. Iterative solution-seeking is necessary, and parameter adjustments are implemented to address these complexities. Therefore, by defining the reference $\Lambda_{f}=\left(x_{d},y_{d},\varphi_{d}\right)$ and error item *e*, we can consider the cost function as follows:

(12) $$J(e\left(t_{k}\right),u_{f})=\int_{t_{k}}^{t_{k}+T}L(e\left(\tau \right),u_{f})d\tau +g\left(e\left(t_{k}+T\right)\right),$$

where *L* indicates the stage cost while *g* is the terminal penalty. *T* is the prediction horizon. Furthermore, the optimal control problem addressed at each sampling instant can be structured as follows:

(13) $$\min_{u_{f}}J(e\left(t_{k}\right),u_{f}),$$

Subject to

(14) $$e\left(t_{k}\right)=\xi (x\left(t_{k}\right),y\left(t_{k}\right),\varphi \left(t_{k}\right),R_{f}),$$

(15) $$L=e{\left(\tau |t_{k}\right)}^{T}Qe\left(\tau |t_{k}\right)+u_{f}{\left(\tau |t_{k}\right)}^{T}Ru_{f}\left(\tau |t_{k}\right),$$

(16) $$g=e{\left(t_{k}+T|t_{k}\right)}^{T}Ke\left(t_{k}+T|t_{k}\right),$$

(17) $$u_{f}\in \left[u_{f\min},u_{f\max}\right],$$

where ξ can be calculated by the motion model of the robotic fish. *Q*, *R*, and *K* represent the coefficient matrix. Through the resolution of the optimization problem outlined above, the optimal control sequence can be derived. Subsequently, solely the control sequence up to the next sampling instant is considered, and the optimization process is reiterated in a receding horizon way.

## 4. Simulation and Analysis

In this section, extensive simulation tests were conducted to validate the effectiveness of the proposed target following method. Firstly, we constructed a simulated pool environment and performed network training using Pytorch 1.12.1. In pursuit of real-time performance, a four-layer fully connected structure was chosen for the architecture of the Actor and Critic networks. The neuron counts in the intermediate two hidden layers are set to 400 and 300, respectively. For key training parameters, the discount factor was set to 0.9, the learning rate to 1 × 10^−5^, the target smoothing coefficient to 0.005, the minimum batch size to 256, the maximum training episodes to 3000, and the maximum training steps per episode to 300. The control period is 0.1 s. The other key parameters of the motion model and control system can be seen in more detail in Table 1.

### 4.1. Training Results and Analysis

**Figure 3:**
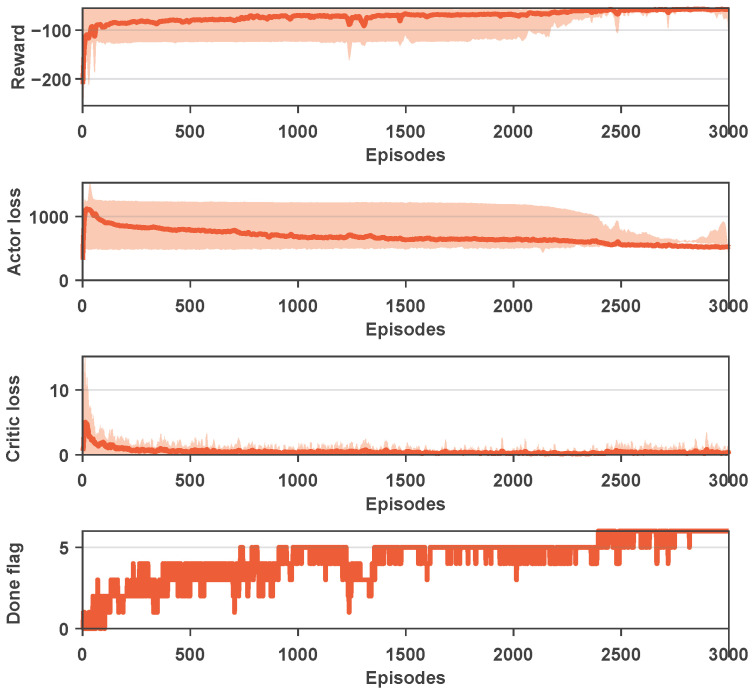
The training results of PDDPG when $N_{p}=5$ .

In this section, the neural network constructed based on the proposed reinforcement learning method was trained. When the prediction step size was set to five, Figure 3 presented the results of six training sessions, including rewards, Actor loss, and Critic loss. The shaded area in the figure represents the range of the maximum and minimum values for each round in the six training sessions. The obtained results indicate that the proposed method exhibits a relatively rapid convergence rate in the initial stages, achieving preliminary convergence at around 500 steps. By the time the training steps reach 3000, complete convergence is achieved, resulting in satisfactory training outcomes.

It should be noted that in Figure 3, the Done flag is used to indicate whether the target point is reached in each training round. In each training session, if the target point was reached within the current round, the variable was set to 1; otherwise, it was set to 0. The Done flag represents the cumulative sum of these binary values in the six training sessions, with a minimum value of zero and a maximum value of six. Therefore, the results suggest that with an increase in the number of training steps, the Done flag exhibited an overall upward trend, indicating that convergence was ultimately achieved in each training session. As a whole, from the reward and loss results, it can be seen that the values reached a satisfactory level after 3000 iterations, and the curves appeared relatively smooth. In terms of completion, the Done flag was essentially at the maximum value around 3000 steps, indicating successful attainment of the target point in each training instance.

**Figure 4:**
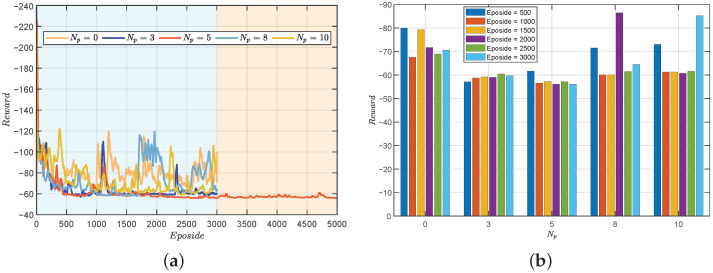
The reward comparison of testing results. (**a**) Under different $N_{p}$ . (**b**) Under different episodes.

To investigate the impact of different prediction steps on training outcomes, we conducted relevant simulation experiments. Initially, we set the parameter $N_{p}$ to values of 0, 3, 5, 8, and 10, employing identical training parameters. The training results are illustrated in Figure 4a. The findings indicate optimal training performance when $N_{p}$ is set to 5, followed by $N_{p}$ = 3. Notably, with $N_{p}$ = 5, not only did rapid convergence occur, but a certain degree of training stability was also observed. Subsequently, we performed an extension of training for the case where $N_{p}$ was set to 5, reaching 5000 steps. The results demonstrate that the reward has stabilized without significant fluctuations.

Further analysis is presented in Figure 4b, depicting comparative results of training with fixed steps under different $N_{p}$ . Overall, PDDPG exhibits superior training performance compared to traditional DDPG, with $N_{p}$ = 3 and $N_{p}$ = 5 showcasing particularly outstanding results. However, it is noteworthy that temporary divergence phenomena were observed during the training processes with $N_{p}$ = 8 and $N_{p}$ = 10. This suggests that when the prediction step size is too small or too large, the training outcomes are unsatisfactory. This phenomenon can be attributed to two main factors. Firstly, a too small prediction interval implies an ineffective restriction on overshooting, leading to frequent changes in system attitude and triggering substantial penalties. Secondly, influenced by the inaccuracy of the motion model, longer prediction intervals may render the system more sensitive to model uncertainty, since model predictions over extended periods may accumulate errors. This could result in a decrease in the robustness of control performance, particularly in the presence of uncertainty or environmental changes.

Hence, the selection of an appropriate prediction interval is crucial for enhancing model training effectiveness, as both excessively small and large prediction intervals may impact the stability and performance of training results. The obtained results support the selection of $N_{p}$ as 5 during the training process, as it exhibits superior performance in terms of rapid convergence and training stability. These findings provide valuable insights into the optimization of prediction step parameters for effective model training.

### 4.2. Testing Results and Analysis

#### 4.2.1. Fix-Point Target Following under Single Robotic Fish

**Figure 5:**
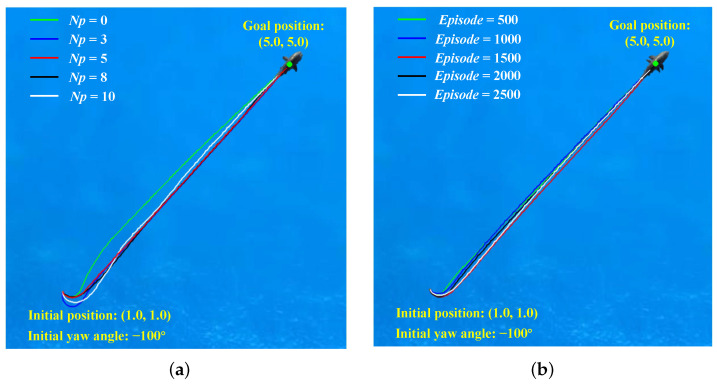
The trajectories comparison of testing results. (**a**) Under different prediction horizons. (**b**) Under different episodes.

**Figure 6:**
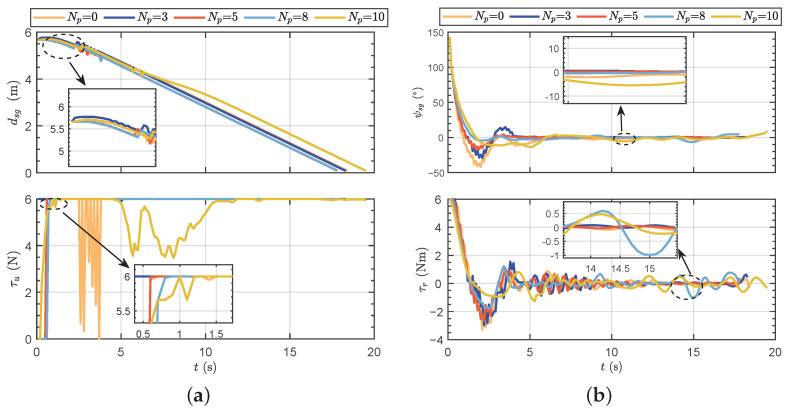
The motion data results of testing results. (**a**) Distance to the target point and forward thrust. (**b**) Yaw angle difference and yaw moment.

To further validate the effectiveness of the proposed method, simulation tests were conducted in this section. Firstly, fix-point target following tests under single robotic fish were performed by setting the initial position, initial attitude, and target point. The trained network results were evaluated based on different prediction horizons. Figure 5a illustrates the motion trajectories of the robotic fish under different prediction horizons. Figure 5b depicts the motion trajectories of the robotic fish at different episodes when $N_{p}$ = 5. Motion data results, including distance to the target point, the yaw angle difference, forward thrust, and yaw moment, are presented in Figure 6.

It can be seen that the trajectories reveal noticeable overshoot phenomenon in the early stages, when the initial pose set at −100°. Without a prediction horizon, the control performance exhibits poor performance. As the prediction horizon increases, the overshoot is effectively suppressed. However, when the prediction horizon reaches 8, some motion instability phenomena begin to emerge. Particularly in the case of $N_{p}=10$ , the yaw angle curve is unsmooth; the reason for this may be that the increased prediction horizon can lead to a chaotic learning process. Specially, although the performance of $N_{p}=3$ is superior to $N_{p}=8$ from a training perspective, the motion trajectories suggest that the performance of $N_{p}=8$ seems more favorable. The reasons for this phenomenon may be identified in Figure 6b. It can be observed that the test results for $N_{p}=8$ show small oscillations in the yaw moment even after entering the steady-state following process, indicating an instability in its swimming posture. The obtained results indicate that there are interactions between the prediction horizon and control performance, and it also influences overshoot suppression and post-steady-state stability in following motion.

#### 4.2.2. Dynamic Target Following under Single Robotic Fish

To assess the effectiveness of the proposed methods in dynamic target following, a standard circle was employed for testing, establishing a foundation for collaborative following. Initially, the circle’s center was positioned at (4, 4) with a set radius of 2. The circle was then partitioned into 200 points and followed sequentially. The robotic fish transitioned to the next target point when its distance to the target point fell below 0.3 m.

**Figure 7:**
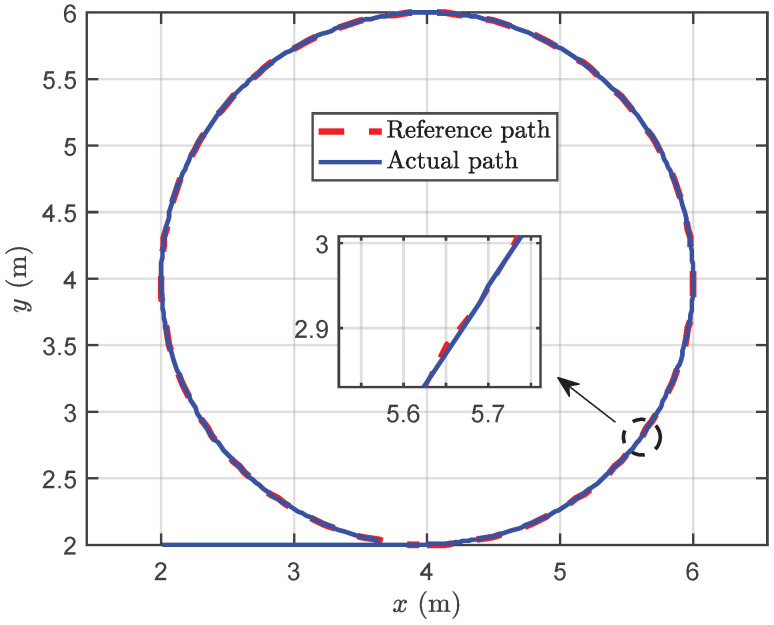
The simulation results of circle following trajectory.

**Figure 8:**
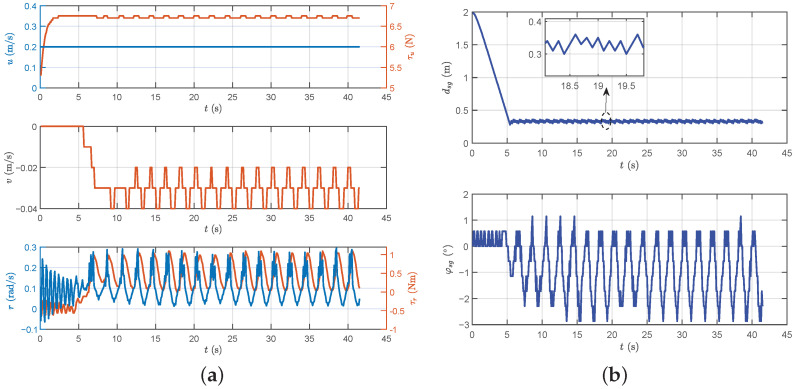
The motion data results of dynamic target following control. (**a**) The velocity illustration and control force/moment. (**b**) The following distance and yaw attitude.

Figure 7 presents the following path, while Figure 8a,b provide insights into the following data. The results demonstrate the successful implementation of the proposed method for standard circle following, yielding some conclusions. Initially, during the stable following process, the distance between the robotic fish and the dynamic target point is maintained at 0.32 m. According to the switch criterion for dynamic target following, the proposed method can be validated to accomplish following promptly and effectively. Subsequently, the yaw attitude during the following process exhibits relative stability but with subtle oscillations. The forward thrust remains at its maximum value for the majority of the time, which is primarily attributed to the consistent distance between the robotic fish and the dynamic target point. In the context of turning motion, a certain degree of lateral movement is induced, resulting in a lateral velocity. Finally, consistent with the performance of yaw attitude, both the yaw moment and turning angular velocity display slight oscillations characterized by small amplitudes, without significantly affecting the system stability.

#### 4.2.3. Cooperative Following Control under Multiple Robotic Fish

In this section, inspiration from the efficient mechanisms of fish schooling motion is applied to multi-robot cooperative motion. Studies indicate that maintaining a certain distance and direction between multiple fish swimming together can enhance hydrodynamic performance. For instance, swimming in a side-by-side following manner can reduce energy consumption. Therefore, we emulate the principles of fish swarm motion at the locomotion level, laying the foundation for research on robotic fish swarms. It should be noted that the proposed methods in this paper can be directly applied to any agent in the cluster, and are not limited to the number of agents. To better demonstrate simulation results, this section takes the example of a following task with two biomimetic robotic fish to validate the effectiveness of the proposed methods.

Throughout the cooperative following involving multiple robotic fish, the dynamic repositioning of the leader robotic fish prompts a corresponding adjustment in the target-following coordinates $P_{f}^{i}$ for the follower robotic fish. Hence, if the initial position of the follower robotic fish remains fixed, $d_{\max}$ is a variable which will introduce a notable drawback. In the scenario where the leader robotic fish is engaged in a single-point following task, the ongoing following motion results in a progressive increase in the distance $P_{f}^{i}$ from the initial point, leading to an augmentation of the follower robotic fish’s $d_{\max}$ , as indicated in Equation (9). Despite the continuous pursuit of the leader by the follower robotic fish, it is indicated that there is potential for a decreasing $d_{\max}$ , ultimately causing a gradual reduction in pursuit speed until it becomes insufficient for successful following. To mitigate this challenge, both $d_{\max}$ for the leader and follower robotic fish are intentionally maintained as constants, specifically set at 6 during the testing phase in this section.

Furthermore, we outline a task for a biomimetic autonomous system. The task involves a leader robotic fish guiding a collective of follower robotic fish in the exploration of a predefined area. The exploration process is facilitated by establishing search target points for the leader robotic fish, with the other follower robotic fish collaboratively tailing the leader in the exploration endeavor. Besides, the distances and orientations during the following process can be determined based on biological mechanisms observed in natural fish. Consequently, this section presents the simulation testing of leader–follower cooperative following control. First, the corner points of a square are defined as search target points for the leader robotic fish, specifically at (6, 2), (6, 6), (2, 6), and (2, 2). The leader robotic fish aligns its movement towards these target points, switching to the next target point when the distance from the current target falls below 0.3 m. Further, the follower robotic fish dynamically follows the movement of the leader robotic fish in real time.

**Figure 9:**
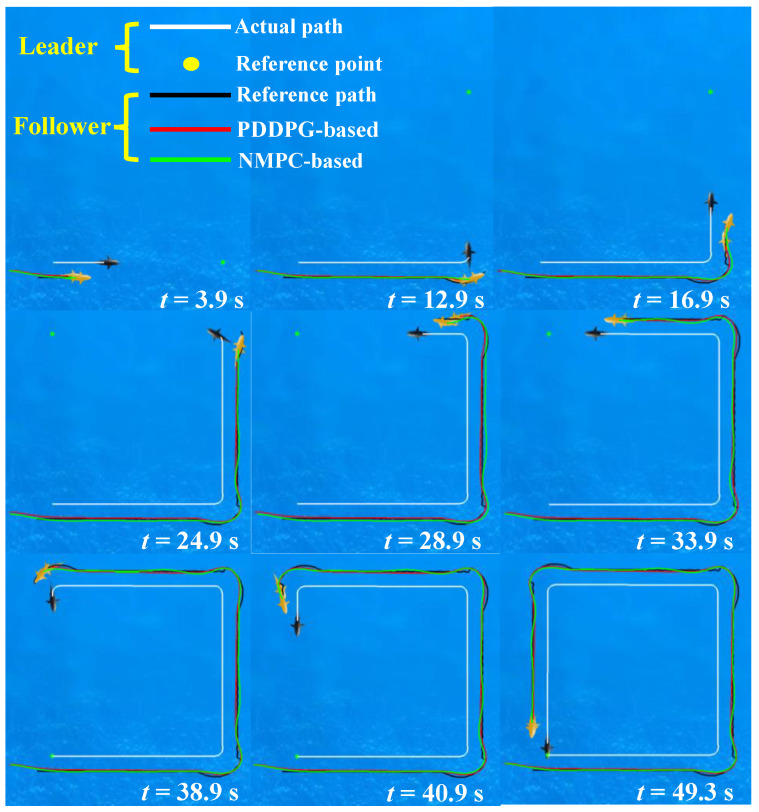
The snapshot sequences of cooperative following control.

In alignment with the biological mechanisms of collaborative motion [16], we stipulate a distance of $d_{f}^{i}=0.5$ m and a target orientation of $\varphi_{f}^{i}=135^{\circ }$ concerning the leader robotic fish. Thus, based on Equation (1), we can derive the real-time target position for the follower robotic fish. Figure 9 provides snapshot sequences of the cooperative following control, encompassing leader trajectory generated by PDDPG, and the follower trajectories generated by PDDPG and NMPC. The obtained results demonstrate that the leader robotic fish successfully completes the standard square path search task with minimal overshooting. Notably, the follower robotic fish, under the control of both PDDPG and NMPC methods, successfully accomplishes the following task.

**Figure 10:**
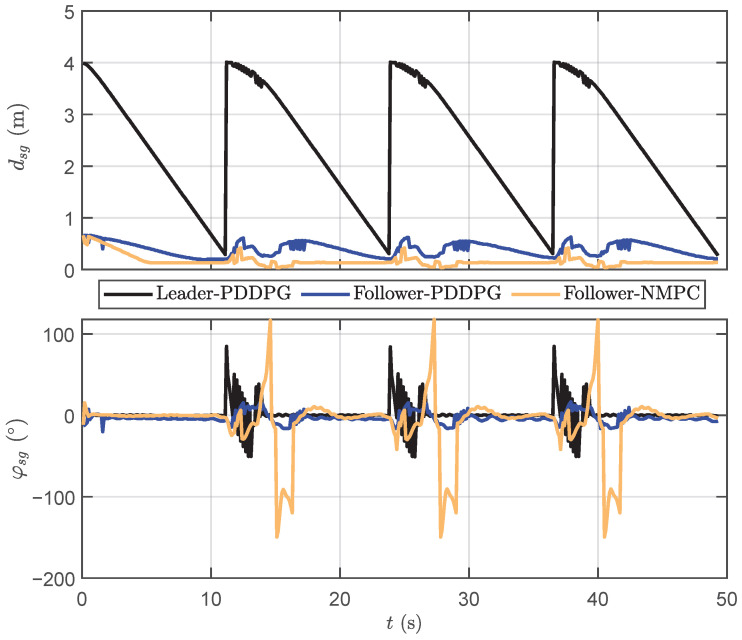
The motion data results of cooperative following distance and yaw difference.

Figure 10 depicts the motion data results for cooperative following. Based on $d_{sg}$ , it can be seen that NMPC displays a marginally superior performance in following distance compared to PDDPG, indicating a capacity for faster target following. However, concerning $\varphi_{sg}$ , PDDPG significantly outperforms NMPC, especially when the leader robotic fish switches target points for a right-angle turn. The distinct weak overshooting characteristic of PDDPG is conspicuously manifested, while NMPC exhibits a noticeable degree of overshooting, resulting in unstable yaw attitude. To further verify the superiority of the proposed method, some quantitative comparison results are offered. On one hand, the total lengths of the following path generated by PDDPG and NMPC are 18.34 m and 18.36 m, respectively, which indicates the slim margin for PDDPG. On the other hand, when the robotic fish turns at a right angle, the overshoot phenomenon is obvious. Taking the time interval of *t* = [10 s, 20 s] as an example, the root-mean-square error (RMSE) of $\varphi_{sg}$ for PDDPG and NMPC are $8.3^{\circ }$ and $45.6^{\circ }$ , respectively. Additionally, the mean absolute error (MAE) of $\varphi_{sg}$ for PDDPG and NMPC are $7^{\circ }$ and $25.5^{\circ }$ , respectively. Therefore, the obtained results illustrate that the proposed PDDPG shows more satisfactory performance.

**Figure 11:**
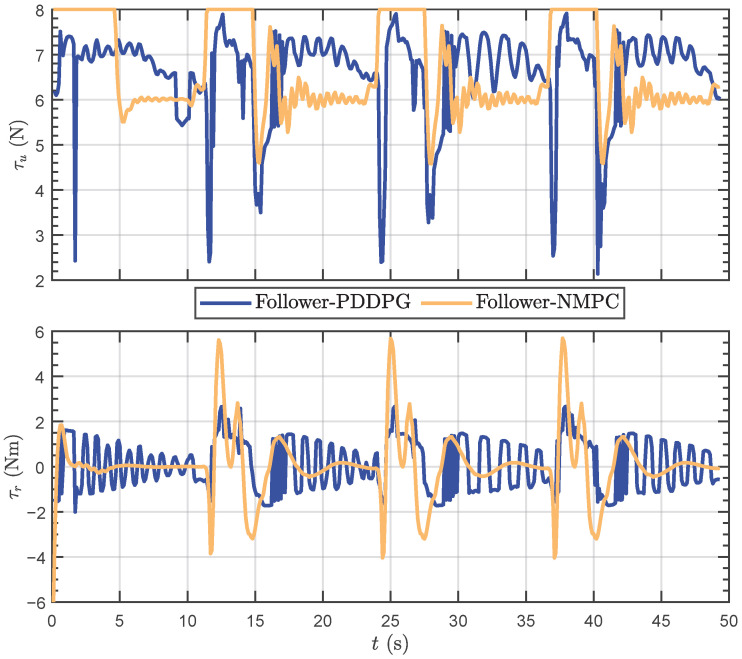
The motion data results of control quantities.

Moreover, Figure 11 provides insight into the control quantities of forward thrust and yaw moment. In terms of forward thrust, PDDPG seldom reaches its maximum value, whereas NMPC consistently maintains a near-maximum swimming speed throughout the pursuit process. This may be attributed to PDDPG learning, which revealed that that overshooting is prone to occur during high-speed turns, prompting the model to avoid utilizing maximum thrust to prevent this phenomenon. Regarding yaw moments, consistent with yaw attitudes during turning, NMPC outputs a more substantial moment amplitude during turning phases. Nevertheless, it can be observed that in the steady-state tracking phase, NMPC’s yaw moment is more stable, while PDDPG exhibits slight oscillations, resembling the swimming behavior of real tail-flapping fish.

### 4.3. Discussion

Drawing inspiration from the schooling movement of biological fish, a target-following method based on deep reinforcement learning is proposed, leading to successful implementation of cooperative following control. On one hand, by incorporating predictive thinking into the traditional DDPG algorithm, the system overshooting is effectively reduced. Notably, the method utilizes static target-following scenarios during training but demonstrates the ability to follow dynamic targets. On the other hand, as an auxiliary control, a target-following controller based on NMPC is designed.

When adjusting the parameters of neural networks and hyperparameters of deep reinforcement learning, we adhere rigorously to the principle of cross-validation to identify the most suitable parameter combinations for a specific task while mitigating issues such as overfitting or underfitting. With the consideration of model complexity, we explore multiple combinations of neurons, conduct cross-validation, and ultimately select appropriate parameters. With regard to the discount factor, learning rate, smoothing coefficient, and batch size, we conduct preliminary tests based on conventional DDPG parameters and further engage in simulation testing to finalize the parameters. Concerning certain parameter settings in the reward function of deep reinforcement learning, we adjust the parameters from the aspects of objectives significance and simulation test results. Regarding generalization performance, we employed several strategies to enhance generalization, including the normalization and scaling technique for the state variables, adding the noise for action generation, and adjusting the target network update frequency. Further, in dynamic target following and cooperative following control simulations, even with real-time changes to the target point, the proposed method consistently demonstrated effective following capabilities, providing further evidence of its robust generalization performance.

Furthermore, extensive simulations are conducted. First, the training results reveal optimal performance with a prediction step of $N_{p}=5$ . Excessively large or small prediction periods yield unsatisfactory performance. This conclusion is further validated through stationary target-following tests. Second, to assess dynamic target-following performance, the proposed PDDPG algorithm successfully follows a circular trajectory. Finally, by setting up a cooperative following task, the proposed method accomplishes cooperative exploration in a quadrilateral environment, concluding with a performance comparison between PDDPG and NMPC, confirming the effectiveness and superiority of the proposed method.

Despite the successful implementation of cooperative following control, there are still some limitations. On one hand, this paper places particular emphasis on the motion of individual robotic fish, with the goal of directly transferring learned target-following capabilities to swarm control. Consequently, considerations such as inter-swarm motion constraints or obstacle avoidance are omitted. By incorporating neighboring robotic fish states into the state space and devising appropriate reward functions, this issue may be addressed. On the other hand, we focus on the kinematic level of biological fish schooling movement, without delving into behavioral level [30]. To this end, it is required that the joint dynamic models and biomimetic motion control algorithms should be introduced, which is our ongoing endeavor.

## 5. Conclusions and Future Work

In this paper, we have developed a target-following control framework, including a predictive deep deterministic policy gradient controller and a nonlinear model predictive controller. Inspired by the mechanism of hydrodynamic efficiency improvement observed in fish schooling movement, we aim to investigate a target following method that can be applied to achieve a cooperative following task. In view of the nonlinear characteristics in the motion model of the robotic fish, the predictive modeling concept is incorporated into the conventional DDPG algorithm. On this basis, the training framework is developed, including the normalization of the state space, action space, and the standardization of the reward function. Additionally, we introduce an auxiliary controller based on a nonlinear predictive model, offering an alternative for cooperative following control of the follower robotic fish. Finally, extensive simulations are conducted, demonstrating the effectiveness of the proposed method.

In future work, we plan to further investigate the mechanistic aspects of the behavioral level in the biological fish schooling movement. By incorporating inter-cluster motion constraints, more intelligent cooperative following can be achieved. Furthermore, how to realize three-dimensional cooperative following control is another issue worthy of in-depth study.

## Figures and Tables

**Table 1:** Parameters of the following control system.

Item	Value	Item	Value	Item	Value
$m_{11}$	9.9 kg	$m_{22}$	14.5 kg	$m_{33}$	1.8 kg
$d_{11}$	17.2 kg/s	$d_{22}$	19.3 kg/s	$d_{33}$	1.1 kg·m/s^2^
*Q*	diag{ 50, 50, 0.2}	*R*	diag{ 0.005}	*K*	diag{ 0.5}
$c_{1}$	0.4	$c_{2}$	0.4	$c_{3}$	0.2
$k_{1}$	10	$k_{2}$	20	*T*	10
$u_{f\min}$	$\tau_{u}=0,\tau_{r}=-6$	$u_{f\max}$	$\tau_{u}=8,\tau_{r}=6$		

## Data Availability

The data generated during the current study are available from the corresponding author on reasonable request.
